# Inhibition of USP11 attenuates sepsis-associated acute kidney injury by downregulating TGFBR2/Smad3 signaling

**DOI:** 10.3389/fmolb.2025.1571593

**Published:** 2025-06-27

**Authors:** Lu Wang, Wen Tang, Long Jiang, Daquan Zhang, Zhigao Wang, Rennan Guo, Jingjing Wang, Dong Xiao

**Affiliations:** Department of Critical Care Medicine, People’s Hospital of Xinjiang Uygur Autonomous Region, Urumqi, Xinjiang, China

**Keywords:** USP11, sepsis, acute kidney injury, TGFBR2, Smad3

## Abstract

**Introduction:**

Sepsis-associated acute kidney injury (AKI) is a common complication of sepsis, which is a severe inflammatory disease with high mortality. The TGF-β/Smad signaling pathway plays an important role in the progression of sepsis, and targeting the TGF-β receptor II (TGFBR2) has been shown to ameliorate its effects. Ubiquitin-specific peptidase 11 (USP11) stabilizes TGFBR2 and enhances the TGF-β/Smad signaling pathway. In this study, we evaluated the effects of USP11 inhibition on sepsis-associated AKI.

**Methods:**

A septic mouse model was established and treated with the USP11 inhibitor mitoxantrone. The expression of TGFBR2, phosphorylation of Smad3, as well as the levels of kidney injury markers, inflammatory cytokines, and oxidative stress markers, were measured in kidney tissues.

**Results:**

Elevated expressions of TGFBR2 and phosphorylated Smad3 were detected in the kidneys of septic mice, and mitoxantrone treatment was found to reduce the expression of TGFBR2 while suppressing the activation of Smad3. The drug also attenuated kidney injury while reducing inflammation and oxidative stress in the kidneys of septic mice.

**Conclusion:**

USP11 inhibition by mitoxantrone ameliorated sepsis-associated AKI by downregulating TGFBR2/Smad3 signaling.

## Introduction

Sepsis is a severe inflammatory disease caused by infection or injury and is one of the leading causes of death worldwide (Guarino et al., 2023). Sepsis can induce septic shock and organ dysfunction, with acute kidney injury (AKI) being one of the more serious complications of sepsis (Zarbock et al., 2023). Sepsis-associated AKI has been linked to higher mortality, increased rate of disability, and decreased quality of life in patients (Zarbock et al., 2014; Stanski et al., 2020). Thus, it is critically important to explore the pathogenesis and find targets for effective early diagnosis and treatment of sepsis-induced AKI (He et al., 2022).

Systemic inflammation is the fundamental pathophysiological feature of sepsis (Hotchkiss et al., 2016). Here, sepsis is initiated upon host recognition of certain pathogen-associated or damage-associated molecular patterns and is characterized by activation of the inflammatory signaling pathways. The activation of these signaling cascades can induce expression of factors involved in inflammation and metabolism, ultimately resulting in organ damage. The overwhelming production of proinflammatory cytokines, such as interleukin (IL)-1β, IL-6, tumor necrosis factor (TNF)-α, as well as reactive oxygen species (ROS) is a hallmark of sepsis and sepsis-associated AKI. Targeting these inflammatory cytokines and oxidative stress may help ameliorate sepsis-associated AKI (Al-Harbi et al., 2019; Xia et al., 2019; Al-Kadi et al., 2022; Chen et al., 2023).

The transforming growth factor-β (TGF-β) signaling pathway contains multiple cytokines and mediates many aspects of embryogenesis and tissue homeostasis (Tzavlaki and Moustakas, 2020). TGF-β signaling is initiated by the binding of TGF-β to the type II (TGFBR2) and type I (TGFBR1) receptors on the cell membrane, which results in phosphorylation and activation of TGFBR1 by TGFBR2 (Massague and Chen, 2000). The activated TGFBR1 phosphorylates Smad2/3 for downstream gene activation. Ubiquitination has been shown to degrade the TGF-β receptors and regulate the TGF-β signaling pathways (Huang and Chen, 2012). The ubiquitination system includes the substrate, ubiquitin, ubiquitin-activating enzymes (E1), ubiquitin conjugating enzymes (E2), ubiquitin ligase enzymes (E3), and deubiquitinating enzymes (DUBs) (Nandi et al., 2006; Snyder and Silva, 2021). Ubiquitin-specific peptidase 11 (USP11) has been shown to promote TGF-β signaling through stabilization of TGFBR2, and inhibition of USP11 increases the ubiquitination of TGFBR2 to promote its degradation (Jacko et al., 2016; Garcia et al., 2018b; Ni et al., 2023). Elevated TGF-β and TGFBR2 levels have been detected in patients with sepsis, suggesting the critical role of TGF-β/Smad signaling in sepsis (Marie et al., 1996; Huang et al., 2010). Ma et al. (2019) revealed that miRNA-145 targets TGFBR2; accordingly, the knockdown of TGFBR2 or overexpression of miRNA-145 can attenuate lipopolysaccharide (LPS)-induced sepsis to improve the survival rates of septic mice, suggesting that TGFBR2 could be a potential target for treating sepsis (Ma et al., 2019). Herein, we hypothesize that inhibiting USP11 activity could regulate TGFBR2 in sepsis and provide protection against sepsis-associated AKI.

## Materials and methods

### Mouse model of sepsis

In the present study, sepsis was induced in mice using the cecal ligation and puncture (CLP) method, as described in a previous work (Jin et al., 2022). The mice were anaesthetized via intraperitoneal injection of 60-mg/kg pentobarbital and placed on a heating blanket to maintain the body temperature. Then, approximately one-third of the cecum was ligated using a 5-0 suture and punctured twice using a 21-gauge needle. The mice in the sham group underwent an identical procedure except for ligation and puncture. Samples were collected from the kidney and other tissues at various time points after CLP. The mice then received an intravenous dose of 1, 3, or 5 mg/kg mitoxantrone (MCE, Shanghai, China; cat. no. HY-13502) via penile vein injection. All mouse studies were approved by the People’s Hospital of Xinjiang Uygur Autonomous Region.

### Western blotting

The mouse kidneys were homogenized and total protein was extracted using sodium dodecyl sulfate (SDS) lysis buffer (Beyotime, Shanghai, China; cat. no. P0013G). Then, equal amounts of the total protein (30 μg per group) were subjected to SDS-PAGE and Western blotting following standard protocols. The primary antibodies used in this study were as follows: anti-TGFBR2 (Abcam, 1:1000; cat. no. ab259360), anti-TGFBR1 (Abcam, 1:1000; cat. no. ab235578), anti-Smad3 (Abcam, 1:1000; cat. no. ab208182), anti-phosphorylated Smad3 (CST, 1:1000; cat. no. 9520), anti-USP11 (Abcam, 1:2000; cat. no. ab109232), anti-fibronectin (Abcam, 1:2000; cat. no. ab2413), anti-α-smooth-muscle actin (anti-α-SMA; Sigma, 1:1000; cat. no. A2547), and anti-GAPDH (Abcam, 1:2000; cat. no. ab8245). The band densities were analyzed using ImageJ software.

### Quantitative real-time polymerase chain reaction (qRT-PCR)

The total RNA was extracted using TRIzol reagent (Thermo Fisher; cat. no. 15596018CN), and cDNA was synthesized using the RevertAid First Strand cDNA Synthesis Kit (Thermo Fisher; cat. no. K1622). Next, qRT-PCR was performed using SYBR Green qPCR Master Mix (Thermo Fisher; cat. no. 4309155) on a QuantStudio5 real-time PCR system (Thermo Fisher). The primers sequences were as follows: *Tgfbr2*: 5′-ACGTTCCCAAGTCGGATGTG-3’ (forward) and 5′-ACAGCTTAGGTGGATGGATGC-3’ (reverse); *Usp11*: 5′-CGTTTCCGGGACCAGAATCC-3’ (forward) and 5′-CATCGCCGTCCGTTCTCTTC-3’ (reverse); *GAPDH*: 5′-CTTTGGTATCGTGGAAGGACTC-3’ (forward) and 5′-AGTAGAGGCAGGGATGATGT-3’ (reverse). The 2^−ΔΔct^ method was used to calculate the relative expression.

### Kidney function analysis

Blood samples were collected from the mice and incubated at 4°C for 1 h to promote clotting. Subsequently, the samples were centrifuged at 3,000 rpm for 15 min at 4°C. Finally, the upper pale-yellow layer (serum) was carefully collected, and the levels of serum creatinine (Scr; cat. no. C011-2-1), serum cystatin C (ScysC; cat. no. H336-1-2), and blood urea nitrogen (BUN; cat. no. C013-2-1) were measured using commercial kits from Nanjing Jiancheng Bioengineering Institute. The urine levels of kidney injury molecule-1 (KIM-1; cat. no. ab119596) and neutrophil gelatinase-associated lipocalin (NGAL; cat. no. ab199083) were measured using commercial enzyme-linked immunosorbent assay (ELISA) kits from Abcam according to manufacturer protocols.

### ELISA

The kidney tissues were homogenized in ice-cold phosphate-buffered saline (PBS), followed by two consecutive centrifugations at 4°C (5,000×*g* and 15 min) to obtain the clarified supernatants containing proteins. Then, the tissue extracts were prepared for ELISA to assess the levels of TGF-β, IL-6, TNF-α, IL-1β, and monocyte chemotactic protein-1 (MCP-1) using the corresponding kits (TGF-β1, LS Bio, cat. no. LS-F5184-1; IL-6, Abcam, cat. no. ab100713; TNF-α, Abcam, cat. no. ab108910; IL-1β, Abcam, cat. no. ab197742; MCP-1, Abcam, cat. no. ab100722) following the manufacturers’ protocols.

### Oxidative stress detection

The kidney tissues were homogenized, and the protein concentrations were determined by the BCA assay (Abcam, cat. no. ab102536). The ROS level was determined using the 2′,7′-dichlorofluorescein diacetate (DCFH-DA) assay, as described previously (Ji et al., 2019). The malondialdehyde (MDA) level was measured using the Lipid Peroxidation Assay kit (Abcam, cat. no. ab238537), and superoxide dismutase (SOD) level was measured using the corresponding colorimetric activity kit (Thermo Fisher, cat. no. EIASODC). Finally, the catalase (CAT) level was measured using the Catalase Activity Assay kit (Abcam, cat. no. ab83464).

### Statistical analysis

The data were presented as mean ± standard deviation and analyzed using GraphPad Prism software. Student’s t-test was used to compare the differences between two groups, and one-way analysis of variance (ANOVA) was used to compare differences among multiple groups, followed by Tukey’s multiple comparisons test for comparison. When *p*-value was less than 0.05, the difference was considered to be significant.

## Results

### Upregulation of TGFBR2 and USP11 in the renal tissues of septic mice

**FIGURE 1:**
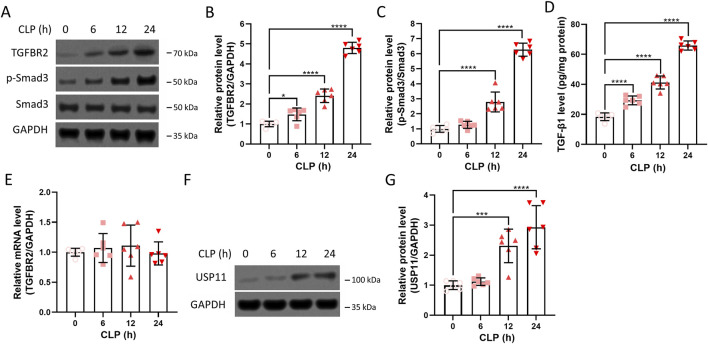
Expressions of TGFBR2 and USP11 in the renal tissues of septic mice. **(A)** Protein expressions of TGFBR2, phosphorylated-Smad3, and Smad3 in kidney tissues were determined by Western blotting, with GAPDH as the loading control, and the optical densities of **(B)** TGFBR2 and **(C)** phosphorylated-Smad3 were analyzed. **(D)** Expression of TGF-β1 in the kidney tissues was determined by ELISA. **(E)** mRNA levels of TGFBR2 in the kidney tissues were determined by qRT-PCR. **(F)** Protein expression of USP11 in the kidney tissues was determined by Western blotting along with the **(G)** optical density of USP11. CLP, cecal ligation and puncture. One-way ANOVA followed by Tukey’s multiple comparison test was used to compare the differences. ^*^ *p* < 0.05, ^***^ *p* < 0.001, and ^****^ *p* < 0.0001.

The expressions of TGFBR2, Smad3, and TGF-β1 in the kidneys were measured after CLP. Increased amounts of the TGFBR2 protein were detected in the kidney, and this increase was time-dependent (Figure 1A). After quantitation, the amounts of TGFBR2 proteins at 6, 12, and 24 h post CLP were significantly higher than that before CLP (Figure 1B), indicating that TGFBR2 was upregulated in the kidneys of septic mice. In contrast, the total amount of Smad3 did not change after CLP, while the amount of phoshoryalted-Smad3 increased significantly in a time-dependent manner (Figures 1A,C). In addition, the expression of TGF-β1 increased in a time-dependent manner (Figure 1D). These results indicate activation of the TGF-β/Smad signaling pathway in the renal tissues of septic mice. We further found that the mRNA level of TGFBR2 remained unchanged after CLP (Figure 1E), suggesting that any changes in the TGFBR2 protein levels were due to the post-transcriptional mechanisms. As USP11 has been described to regulate TGFBR2, we continued to measure the expression of USP11 after CLP. As presented in Figures 1F,G, remarkable increases in the amounts of USP11 protein were detected at 12 and 24 h post CLP. In contrast, CLP did not significantly upregulate TGFBR2 and phoshoryalted-Smad3 in USP11-knockout mice (Supplementary Figures S1, S2). Collectively, these results demonstrate that USP11 may have an important role in sepsis-associated AKI and that inhibiting USP11 may be a promising strategy for the treatment of sepsis-associated AKI.

### Inhibition of USP11 ameliorates kidney injury in septic mice

**FIGURE 2:**
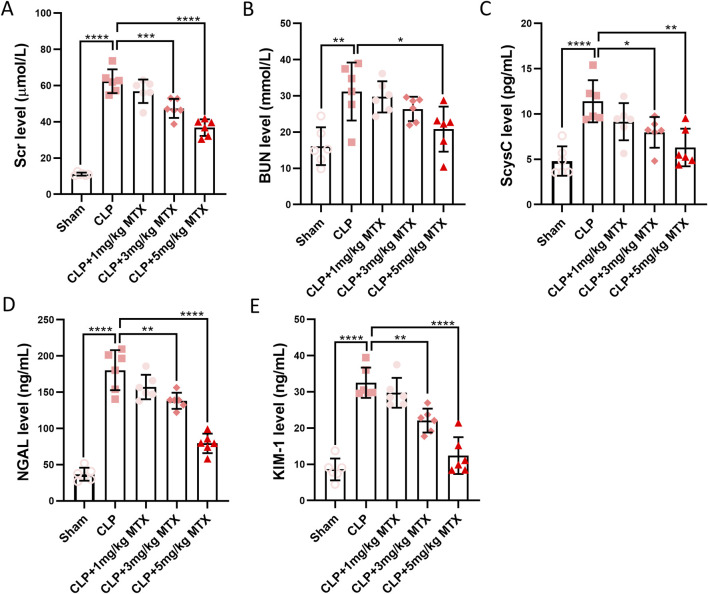
USP11 inhibitor mitoxantrone (MTX) decreases the levels of renal injury biomarkers induced by sepsis; the levels of **(A)** Scr, **(B)** BUN, and **(C)** ScysC were determined using an automatic biochemical analyzer, and the levels of **(D)** NGAL and **(E)** KIM-1 were determined using commercial kits. One-way ANOVA followed by Tukey’s multiple comparison test was used to compare the differences. ^*^ *p* < 0.05, ^**^ *p* < 0.01, ^***^ *p* < 0.001, and ^****^ *p* < 0.0001.

To evaluate the effects of USP11 inhibition on kidney injury, the septic mice were administered the USP11 inhibitor mitoxantrone, and the levels of the kidney function markers Scr, ScysC, BUN, KIM-1, and NGAL were measured. In the septic mice, we detected significantly increased levels of Scr (Figure 2A), BUN (Figure 2B), ScysC (Figure 2C), NGAL (Figure 2D), and KIM-1 (Figure 2E); in contrast, the levels of these markers were significantly lowered in septic mice treated with 5 mg/kg of mitoxantrone, indicating that inhibition of USP11 restored kidney function. In addition, the tubular injury score based on H&E staining is shown in Supplementary Figure S3.

### Inhibition of USP11 suppresses renal inflammation in septic mice

**FIGURE 3:**
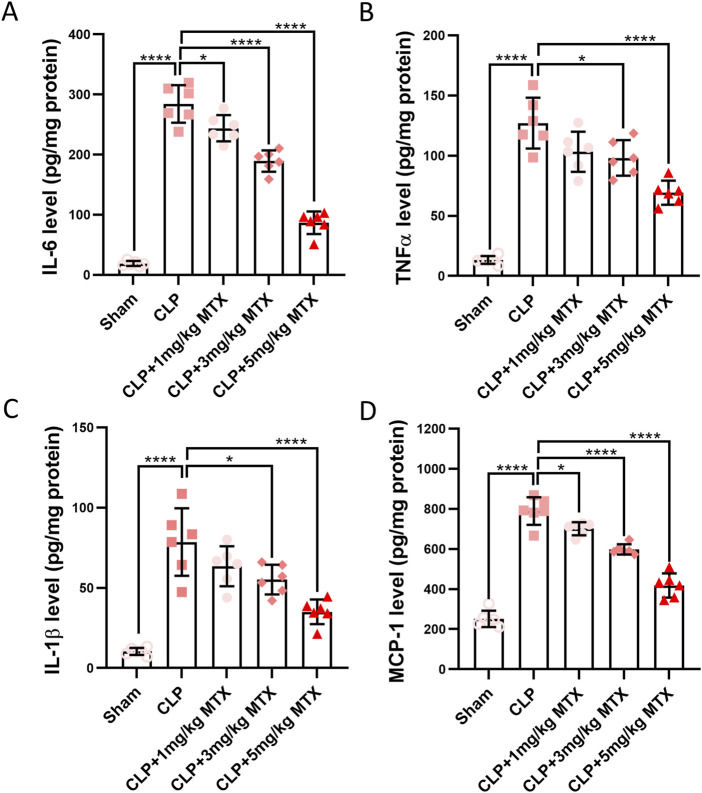
USP11 inhibitor mitoxantrone (MTX) alleviates the expressions of proinflammatory cytokines in the renal tissues of septic mice. The expression levels of inflammatory cytokines in the kidney tissues, including those of **(A)** IL-6, **(B)** TNF-α, **(C)** IL-1β, and **(D)** MCP-1 were determined by ELISA. One-way ANOVA followed by Tukey’s multiple comparison test was used to compare the differences. ^*^ *p* < 0.05; ^****^ *p* < 0.0001.

We continued to evaluate the effects of USP11 inhibition on inflammation in the kidneys of septic mice. Significantly elevated levels of IL-6 (Figure 3A), TNF-α (Figure 3B), IL-1β (Figure 3C), and MCP-1 (Figure 3D) were detected in the kidney homogenates of these mice. In contrast, the levels of these four inflammatory cytokines were remarkably reduced in the kidney homogenates of septic mice treated with 5 mg/kg of mitoxantrone. These results demonstrate that inhibition of USP11 suppressed inflammation in the kidneys of septic mice.

### Inhibition of USP11 suppresses renal oxidative stress in septic mice

**FIGURE 4:**
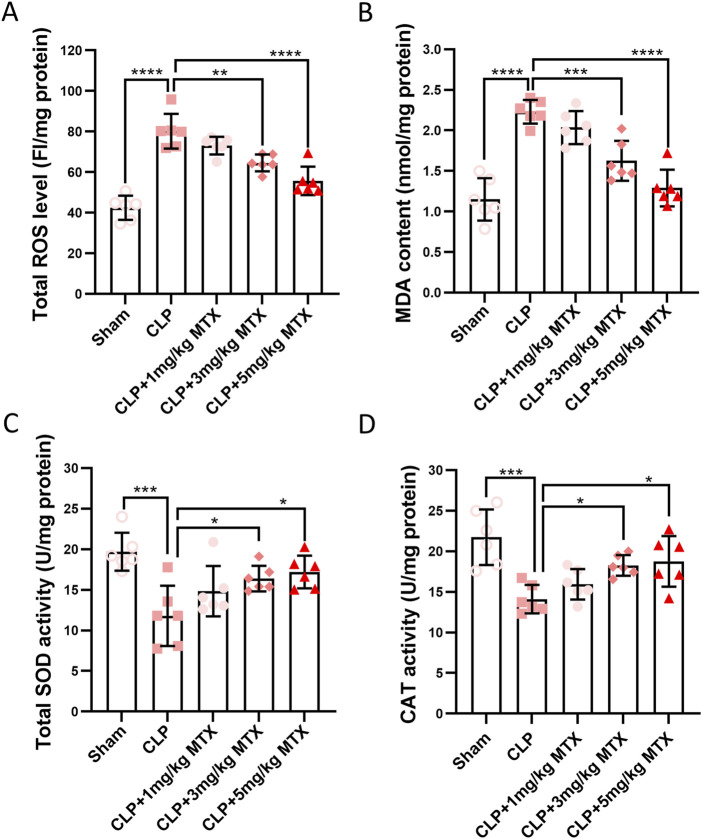
USP11 inhibitor mitoxantrone (MTX) inhibits CLP-induced oxidative stress in mice. The levels of **(A)** total ROS and **(B)** MDA along with the total activities of **(C)** SOD and **(D)** CAT in the kidney tissues were determined. ROS, reactive oxygen species; MDA, malondialdehyde; SOD, superoxide dismutase; CAT, catalase; FI, fluorescence intensity. One-way ANOVA followed by Tukey’s multiple comparison test was used to compare the differences. ^*^ *p* < 0.05, ^**^ *p* < 0.01, ^***^ *p* < 0.001, and ^****^ *p* < 0.0001.

Next, we evaluated the effects of USP11 inhibition on oxidative stress in the kidneys of septic mice. As shown in Figure 4A, the ROS levels were significantly increased in the kidneys of septic mice, while mitoxantrone treatment markedly reduced the ROS levels. Similarly, septic mice exhibited significant increases in renal MDA levels, which were dramatically reduced following mitoxantrone treatment at 5 mg/kg bodyweight (Figure 4B). In septic mice, the levels of antioxidants SOD (Figure 4C) and CAT (Figure 4D) were also significantly decreased, whereas mitoxantrone treatment at 5 mg/kg bodyweight restored both the SOD and CAT levels. Collectively, these results demonstrate that mitoxantrone-mediated inhibition of USP11 effectively suppresses oxidative stress in the kidneys of septic mice.

### Inhibition of USP11 suppresses TGFBR2/Smad3 signaling in septic mice

**FIGURE 5:**
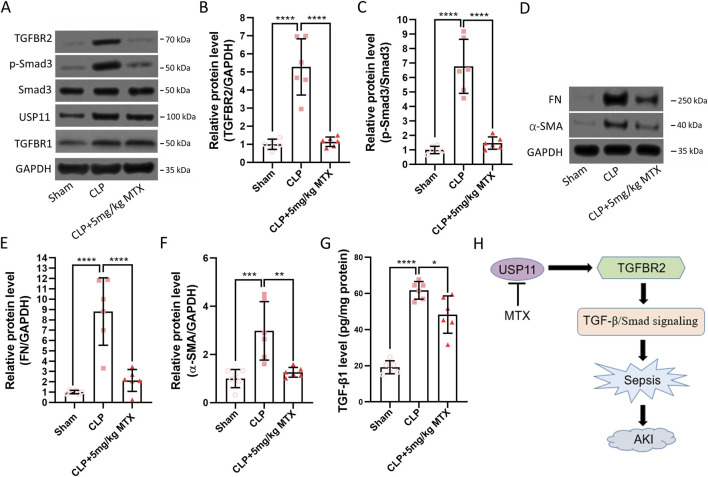
USP11 inhibitor mitoxantrone (MTX) inhibits CLP-induced TGFBR2/Smad signaling in mice. **(A)** Protein expressions of TGFBR2, TGFBR1, phosphorylated-Smad3, Smad3, and USP11 in the kidney tissues were determined by Western blotting, and the optical densities of **(B)** TGFBR2 and **(C)** phosphorylated-Smad3 were analyzed. **(D)** Protein expressions of fibronectin (FN) and α-smooth-muscle actin (α-SMA) in the kidney tissues were determined by Western blotting, and the optical densities of **(E)** FN and **(F)** α-SMA were also analyzed. **(G)** Expression of TGF-β1 in the kidney tissues was determined by ELISA. **(H)** Proposed schematic summary of the study. One-way ANOVA followed by Tukey’s multiple comparison test was used to compare the differences. ^*^ *p* < 0.05, ^**^ *p* < 0.01, ^***^ *p* < 0.001, and ^****^ *p* < 0.0001.

Finally, we evaluated the effects of USP11 inhibition on TGFBR2/Smad3 signaling in the kidneys of septic mice. We detected significantly increased amounts of TGFBR2 (Figures 5A,B) and phosphorylated Smad3 (Figures 5A,C) in the kidneys of septic mice. In contrast, the TGFBR2 and phosphorylated Smad3 levels were markedly decreased in mitoxantrone-treated septic mice. Additionally, mitoxantrone treatment did not affect the expression of USP11 or TGFBR1 (Figure 5A). Correspondingly, elevated levels of TGFBR2/Smad3 downstream factors fibronectin (FN, Figures 5D,E) and α-SMA (Figures 5D,F) were observed in the septic mice, whereas mitoxantrone treatment significantly reduced their expressions. Interestingly, mitoxantrone treatment also reduced the expression of TGF-β1 in the septic mice (Figure 5G). Therefore, it is proposed that USP11 inhibition by mitoxantrone can be used to suppress TGFBR2/Smad3 signaling in septic mice (Figure 5H).

## Discussion

Sepsis is a severe inflammatory disease that can cause multiorgan damage and lead to death (Rhodes et al., 2017). Sepsis-associated AKI is one of the common complications of sepsis, which is associated with increased mortality and reduced quality of life. Owing to limited specific therapeutic options, great efforts are being made to identify potential treatment strategies for sepsis-induced AKI. We found that inhibiting USP11 ameliorated sepsis-associated AKI by regulating TGFBR2, suggesting that USP11 could be a potential therapeutic target for AKI. We demonstrated that TGFBR2 and its downstream factor phosphorylated-Smad as well as USP11 were upregulated in septic mice. Inhibition of USP11 using mitoxantrone suppressed injury, inflammation, and oxidative stress in the kidneys of septic mice. Furthermore, USP11 inhibition by mitoxantrone led to decreased expression of TGFBR2 and suppressed the activation of TGFBR2/Smad3 signaling.

The TGF-β/Smad signaling pathway has been implicated in sepsis, and the elevated TGF-β levels detected in patients with sepsis have been used as prognostic markers (Knapp et al., 1998). TGF-β binds to type II (TGFBR2) and type I (TGFBR1) receptors, resulting in phosphorylation of the Smad proteins and activation of the target genes. We also detected enhanced expressions of TGFBR2 and phosphorylated Smad3, indicating increased activation of the TGF-β/Smad signaling pathway in sepsis. TGFBR2 is regulated by ubiquitination for proteasome degradation. USP11 is a DUB that has been shown to stabilize TGFBR2 expression and enhance TGF-β signaling (Garcia et al., 2018a). In the present study, we detected the increased expression of USP11 in the kidney tissues of septic mice, which was correlated with increased expression of TGFBR2 and enhanced activation of Smad3. Therefore, elevated expression of USP11 is considered to induce increased activation of the TGF-β/Smad signaling pathway.

Targeting the TGF-β/Smad signaling pathway has been shown to ameliorate sepsis. Cao et al. (2019) reported that miRNA-145 directly targets TGFBR2 and that overexpression of miRNA-145 attenuates LPS-induced inflammation and sepsis. Thus, injection of miRNA-145 attenuated LPS-induced inflammation and ameliorated sepsis to improve the survival of septic mice; in contrast, TGFBR2 overexpression was found to partially abolish miRNA-145-mediated suppression of LPS-induced inflammation and sepsis-induced acute lung injury (Cao et al., 2019). In another similar study, Ma et al. (2019) found that miRNA-145 directly targeted TGFBR2 and inactivated the TGFBR2/Smad2 axis; they also found that Smad2 could directly bind to DNA methyltransferase 1 (DNMT1), which hypermethylates the miRNA-145 promoter and downregulates miRNA-145 to promote TGFBR2 expression. Overexpression of TGFBR2 abrogates miRNA-145-mediated inhibition of LPS-induced sepsis. These findings strongly indicate that the TGF-β/Smad signaling pathway could be a potential target for sepsis treatment.

Ubiquitination regulates the TGF-β signaling pathway. It has been shown that TGFBR2 could be ubiquitinated for proteasome degradation, which requires the ubiquitination enzymes E1, E2, and E3 (Huang and Chen, 2012). The ubiquitination process is also precisely regulated by DUBs, which remove ubiquitin from the substrate (Liao et al., 2022). The DUB USP11 has been shown to regulate TGFBR2, stabilize its expression, and enhance downstream signaling of TGF-β (Jacko et al., 2016; Garcia et al., 2018b). Therefore, regulation of USP11 could alter the TGF-β signaling pathway. Mitoxantrone is known as a USP11 inhibitor that inhibits USP11 activity (Istomine et al., 2019). In this study, we demonstrate that mitoxantrone treatment decreases the expression of TGFBR2, suppresses inflammation and oxidative stress, ameliorates AKI, and suppresses Smad3 activation in septic mice. The TGF-β/Smad signaling pathway has been shown to orchestrate both pro- and anti-inflammatory responses (Rustenhoven et al., 2016). Although we detected suppression of inflammation in the kidneys of septic mice after mitoxantrone treatment, the underlying mechanism must be explored further. TGF-β also regulates oxidative stress by increasing ROS production and controlling the antioxidative system (Krstic et al., 2015; Chung et al., 2021). Therefore, downregulation of the TGF-β/Smad signaling pathway by mitoxantrone suppresses oxidative stress in septic mice.

Our study demonstrates the protective effects of USP11 inhibition by mitoxantrone on AKI in mice. Further studies are needed in clinical patients to validate these findings. It would be both interesting and important to confirm upregulation of the TGFBR2/Smad signaling pathway in patients with sepsis. Mitoxantrone was first approved by the United States Food and Drug Administration in 1987 to treat adult acute myeloid leukemia; however, it is now used to treat acute non-lymphocytic leukemia, advanced prostate cancer, and multiple sclerosis as well (Fox, 2004). Therefore, it is reasonable to investigate the potential therapeutic effects of mitoxantrone in patients with sepsis.

## Conclusion

The present study reveals that USP11 is a key regulator of sepsis-induced AKI through modulation of the TGFBR2/Smad3 pathway. CLP-induced sepsis upregulates USP11 and TGFBR2, leading to inflammation, oxidative stress, and renal damage. However, inhibition of USP11 with mitoxantrone significantly improved kidney function, reduced cytokine levels, and suppressed TGFBR2/Smad3 signaling as well as its downstream fibrotic markers. These findings suggest that USP11 inhibition may be a promising therapeutic approach for treating sepsis-associated kidney injury.

## Data Availability

The original contributions presented in this study are included in the article/Supplementary Material, and any further inquiries may be directed to the corresponding author.

## References

[B1] Al-Harbi N. O. Nadeem A. Ahmad S. F. Alanazi M. M. Aldossari A. A. Alasmari F. (2019). Amelioration of sepsis-induced acute kidney injury through inhibition of inflammatory cytokines and oxidative stress in dendritic cells and neutrophils respectively in mice: Role of spleen tyrosine kinase signaling. Biochimie 158, 102–110. 10.1016/j.biochi.2018.12.014 30599182

[B2] Al-Kadi A. El-Daly M. El-Tahawy N. F. G. Khalifa M. M. A. Ahmed A. F. (2022). Angiotensin aldosterone inhibitors improve survival and ameliorate kidney injury induced by sepsis through suppression of inflammation and apoptosis. Fundam. Clin. Pharmacol. 36, 286–295. 10.1111/fcp.12718 34309069

[B3] Cao X. Zhang C. Zhang X. Chen Y. Zhang H. (2019). MiR-145 negatively regulates TGFBR2 signaling responsible for sepsis-induced acute lung injury. Biomed. Pharmacother. 111, 852–858. 10.1016/j.biopha.2018.12.138 30841464

[B4] Chen Y. Wei W. Fu J. Zhang T. Zhao J. Ma T. (2023). Forsythiaside A ameliorates sepsis-induced acute kidney injury via anti-inflammation and antiapoptotic effects by regulating endoplasmic reticulum stress. BMC Complement. Med. Ther. 23, 35. 10.1186/s12906-023-03855-7 36737765 PMC9896724

[B5] Chung J. Huda M. N. Shin Y. Han S. Akter S. Kang I. (2021). Correlation between oxidative stress and transforming growth factor-beta in cancers. Int. J. Mol. Sci. 22, 13181. 10.3390/ijms222413181 34947978 PMC8707703

[B6] Fox E. J. (2004). Mechanism of action of mitoxantrone. Neurology 63, S15–S18. 10.1212/wnl.63.12_suppl_6.s15 15623664

[B7] Garcia D. A. Baek C. Estrada M. V. Tysl T. Bennett E. J. Yang J. (2018a). USP11 enhances tgfβ-induced epithelial-mesenchymal plasticity and human breast cancer metastasis. Mol. Cancer Res. 16, 1172–1184. 10.1158/1541-7786.MR-17-0723 29724812 PMC6030438

[B8] Garcia D. A. Baek C. Estrada M. V. Tysl T. Bennett E. J. Yang J. (2018b). USP11 enhances tgfβ-induced epithelial-mesenchymal plasticity and human breast cancer metastasis. Mol. Cancer Res. 16, 1172–1184. 10.1158/1541-7786.MCR-17-0723 29724812 PMC6030438

[B9] Guarino M. Perna B. Cesaro A. E. Maritati M. Spampinato M. D. Contini C. (2023). 2023 update on sepsis and septic shock in adult patients: management in the emergency department. J. Clin. Med. 12, 3188. 10.3390/jcm12093188 37176628 PMC10179263

[B10] He F. F. Wang Y. M. Chen Y. Y. Huang W. Li Z. Q. Zhang C. (2022). Sepsis-induced AKI: from pathogenesis to therapeutic approaches. Front. Pharmacol. 13, 981578. 10.3389/fphar.2022.981578 36188562 PMC9522319

[B11] Hotchkiss R. S. Moldawer L. L. Opal S. M. Reinhart K. Turnbull I. R. Vincent J. L. (2016). Sepsis and septic shock. Nat. Rev. Dis. Prim. 2, 16045. 10.1038/nrdp.2016.45 28117397 PMC5538252

[B12] Huang F. Chen Y. G. (2012). Regulation of TGF-beta receptor activity. Cell Biosci. 2, 9. 10.1186/2045-3701-2-9 22420375 PMC3333473

[B13] Huang L. F. Yao Y. M. Dong N. Yu Y. He L. X. Sheng Z. Y. (2010). Association between regulatory T cell activity and sepsis and outcome of severely burned patients: a prospective, observational study. Crit. Care 14, R3. 10.1186/cc8232 20064232 PMC2875505

[B14] Istomine R. Alvarez F. Almadani Y. Philip A. Piccirillo C. A. (2019). The deubiquitinating enzyme ubiquitin-specific peptidase 11 potentiates TGF-beta signaling in CD4(+) T cells to facilitate Foxp3(+) regulatory T and T(H)17 cell differentiation. J. Immunol. 203, 2388–2400. 10.4049/jimmunol.1801689 31554694

[B15] Jacko A. M. Nan L. Li S. Tan J. Zhao J. Kass D. J. (2016). De-ubiquitinating enzyme, USP11, promotes transforming growth factor beta-1 signaling through stabilization of transforming growth factor beta receptor II. Cell Death Dis. 7, e2474. 10.1038/cddis.2016.371 27853171 PMC5260874

[B16] Ji Y. Gao Y. Chen H. Yin Y. Zhang W. (2019). Indole-3-Acetic acid alleviates nonalcoholic fatty liver disease in mice via attenuation of hepatic lipogenesis, and oxidative and inflammatory stress. Nutrients 11, 2062. 10.3390/nu11092062 31484323 PMC6769627

[B17] Jin L. Liao W. Zhou X. Wang Y. Qian J. (2022). Hydrocortisone alleviates sepsis-induced acute kidney injury through HSF-1-mediated transcriptional suppression of XPO1. Tissue Cell 79, 101915. 10.1016/j.tice.2022.101915 36087493

[B18] Knapp S. Thalhammer F. Locker G. J. Laczika K. Hollenstein U. Frass M. (1998). Prognostic value of MIP-1 alpha, TGF-beta 2, sELAM-1, and sVCAM-1 in patients with gram-positive sepsis. Clin. Immunol. Immunopathol. 87, 139–144. 10.1006/clin.1998.4523 9614928

[B19] Krstic J. Trivanovic D. Mojsilovic S. Santibanez J. F. (2015). Transforming growth factor-beta and oxidative stress interplay: implications in tumorigenesis and cancer progression. Oxid. Med. Cell Longev. 2015, 654594. 10.1155/2015/654594 26078812 PMC4452864

[B20] Liao Y. Zhou D. Wang P. Yang M. Jiang N. (2022). Ubiquitin specific peptidase 11 as a novel therapeutic target for cancer management. Cell Death Discov. 8, 292. 10.1038/s41420-022-01083-5 35715413 PMC9205893

[B21] Ma F. Li Z. Cao J. Kong X. Gong G. (2019). A TGFBR2/SMAD2/DNMT1/miR-145 negative regulatory loop is responsible for LPS-induced sepsis. Biomed. Pharmacother. 112, 108626. 10.1016/j.biopha.2019.108626 30784922

[B22] Marie C. Cavaillon J. M. Losser M. R. (1996). Elevated levels of circulating transforming growth factor-beta 1 in patients with the sepsis syndrome. Ann. Intern. Med. 125, 520–521. 10.7326/0003-4819-125-6-199609150-00034 8779480

[B23] Massague J. Chen Y. G. (2000). Controlling TGF-beta signaling. Genes Dev. 14, 627–644. 10.1101/gad.14.6.627 10733523

[B24] Nandi D. Tahiliani P. Kumar A. Chandu D. (2006). The ubiquitin-proteasome system. J. Biosci. 31, 137–155. 10.1007/BF02705243 16595883

[B25] Ni J. Y. Wang X. Xie H. Y. Yang N. H. Li J. Y. Sun X. A. (2023). Deubiquitinating enzyme USP11 promotes renal tubular cell senescence and fibrosis via inhibiting the ubiquitin degradation of TGF-β receptor II. Acta Pharmacol. Sin. 44, 584–595. 10.1038/s41401-022-00977-5 36045219 PMC9958121

[B26] Rhodes A. Evans L. E. Alhazzani W. Levy M. M. Antonelli M. Ferrer R. (2017). Surviving sepsis campaign: international guidelines for management of sepsis and septic shock: 2016. Intensive Care Med. 43, 304–377. 10.1007/s00134-017-4683-6 28101605

[B27] Rustenhoven J. Aalderink M. Scotter E. L. Oldfield R. L. Bergin P. S. Mee E. W. (2016). TGF-beta1 regulates human brain pericyte inflammatory processes involved in neurovasculature function. J. Neuroinflammation 13, 37. 10.1186/s12974-016-0503-0 26867675 PMC4751726

[B28] Snyder N. A. Silva G. M. (2021). Deubiquitinating enzymes (DUBs): regulation, homeostasis, and oxidative stress response. J. Biol. Chem. 297, 101077. 10.1016/j.jbc.2021.101077 34391779 PMC8424594

[B29] Stanski N. L. Cvijanovich N. Z. Fitzgerald J. C. Bigham M. T. Wong H. R. Genomics of Pediatric Septic Shock I. (2020). Severe acute kidney injury is independently associated with mortality in children with septic shock. Intensive Care Med. 46, 1050–1051. 10.1007/s00134-020-05940-8 32047942 PMC7677896

[B30] Tzavlaki K. Moustakas A. (2020). TGF-Beta signaling. Biomolecules 10, 487. 10.3390/biom10030487 32210029 PMC7175140

[B31] Xia S. Lin H. Liu H. Lu Z. Wang H. Fan S. (2019). Honokiol attenuates sepsis-associated acute kidney injury via the inhibition of oxidative stress and inflammation. Inflammation 42, 826–834. 10.1007/s10753-018-0937-x 30680694

[B32] Zarbock A. Gomez H. Kellum J. A. (2014). Sepsis-induced acute kidney injury revisited: pathophysiology, prevention and future therapies. Curr. Opin. Crit. Care 20, 588–595. 10.1097/MCC.0000000000000153 25320909 PMC4495653

[B33] Zarbock A. Nadim M. K. Pickkers P. Gomez H. Bell S. Joannidis M. (2023). Sepsis-associated acute kidney injury: consensus report of the 28th Acute Disease Quality Initiative workgroup. Nat. Rev. Nephrol. 19, 401–417. 10.1038/s41581-023-00683-3 36823168

