# Supplementary material for: Inhibition of USP11 attenuates sepsis-associated acute kidney injury by downregulating TGFBR2/Smad3 signaling
Source: Front Mol Biosci. 2025 Jun 27;12:1571593. doi: 10.3389/fmolb.2025.1571593 (PMC12245690; doi:10.3389/fmolb.2025.1571593)
Supplement: Supplementary file 1 [file DataSheet1.pdf]

Supplementary Materials

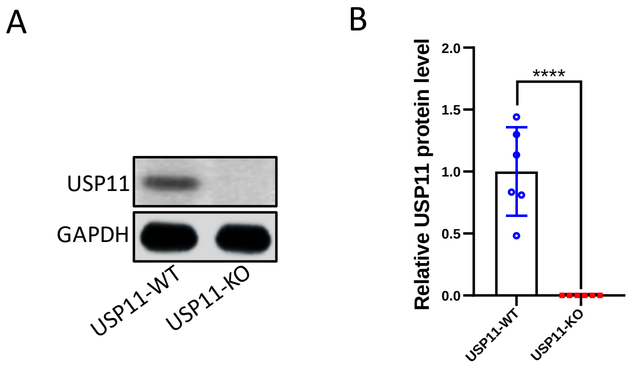

Figure S1. Expression of USP11 in USP11-WT and USP11-KO mice. (A & B) Knockout efficiency of USP11 in the renal tissues of the mice was examined by Western blot (A), and the optical density was also measured (B). WT, wild type; KO, knock out. Student's t test was used to compare differences. $^{****}p$<0.0001.

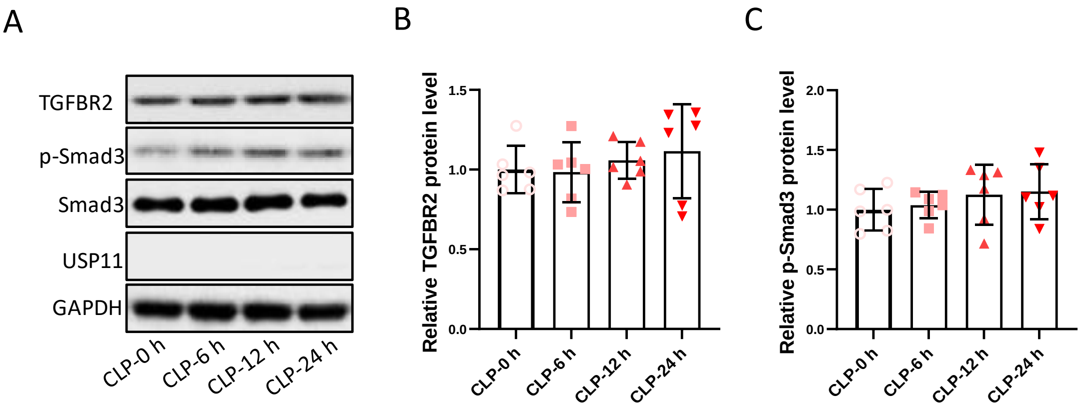

Figure S2. The expression of TGFBR2 and Smad3 in the renal tissue of septic mice with USP11 knockout. (A-C) The protein expressions of TGFBR2, p-Smad3, Smad3 and USP11 in kidney tissues were determined by Western blot (A). GAPDH was used as a loading control. And optical densities of TGFBR2 (B) and p-Smad3 (C) were also analyzed. CLP, cecal puncture; WT, wild type; KO, knock out. One-way ANOVA followed by Tukey's multiple comparisons test was used to compared differences.

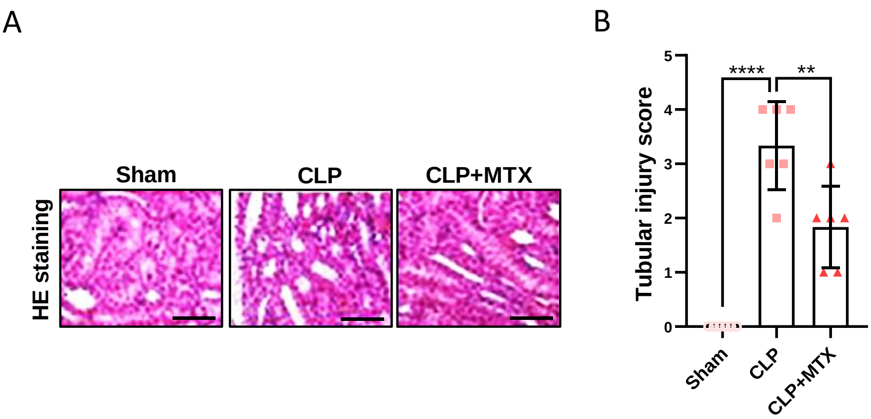

Figure S3. Tubular injury assessed by H & E staining. (A) Tubular injury was assessed by H&E staining, and the representative images were as shown indicated. (B) Tubular injury score based on H&E staining. One-way ANOVA followed by Tukey's multiple comparisons test was used to compared differences. $^{**}p$<0.01, $^{****}p$<0.0001.

Supplementary methods

USP11-knockout mice

USP11-knockout mice were obtained from Cyagen (Suzhou, China) (#S-KO-06854). Mouse genotype was verified through both polymerase chain reaction (PCR) and Western blotting analyses at the mRNA and protein expression levels according to the manufacturer's instruction. All mice were maintained in SPF room.

Histological analysis

Kidney tissues were preserved in 10% formalin, dehydrated, embedded in paraffin, and then sliced into sections with a thickness of 4 micrometers. The extent of acute kidney injury was assessed through hematoxylin and eosin (H&E) staining. Tubular injury in the H&E-stained sections was evaluated semi-quantitatively, based on the proportion of tubules affected, using the following scale: 0 = no damage; 0.5 = less than 12.5% injury; 1.0 = 12.5%–25% injury; 1.5 = 25%–37.5% injury; 2.0 = 37.5%–50% injury; 2.5 = 50%–62.5% injury; 3.0 = 62.5%–75% injury; 3.5 = 75%–87.5% injury; and 4.0 = 87.5%–100% injury. Signs of tubular damage included cell swelling, tubular necrosis and cast formation.
